# Empowering Health Through Digital Lifelong Prevention: An Umbrella Review of Apps and Wearables for Nutritional Management

**DOI:** 10.3390/nu17223542

**Published:** 2025-11-12

**Authors:** Marta Giardina, Rosa Zarcone, Giulia Accardi, Garden Tabacchi, Marianna Bellafiore, Simona Terzo, Valentina Di Liberto, Monica Frinchi, Paolo Boffetta, Walter Mazzucco, Miriana Scordino, Sonya Vasto, Antonella Amato

**Affiliations:** 1 Department of Biological, Chemical and Pharmaceutical Sciences and Technologies (STEBICEF), University of Palermo, Viale delle Scienze, 90128 Palermo, Italy; marta.giardina@unipa.it (M.G.);; 2 Laboratory of Immunopathology and Immunosenescence, Department of Biomedicine, Neurosciences and Advanced Technologies, University of Palermo, Corso Tuköry, 211, 90134 Palermo, Italy; rosa.zarcone@unipa.it; 3 Sport and Exercise Sciences Research Unit, Department of Psychology, Educational Science and Human Movement, University of Palermo, 90144 Palermo, Italy; 4 Department of Biomedicine, Neuroscience and Advanced Diagnostics, University of Palermo, Corso Tukory, 129, 90134 Palermo, Italy; 5 Department of Medicine and Surgery, “Kore” University of Enna, 94100 Enna, Italy; 6 Department of Medical and Surgical Sciences, University of Bologna, Via Massarenti 9, 40138 Bologna, Italy; 7 Department of Health Promotion, Mother and Child Care, Internal Medicine and Medical Specialties, University of Palermo, Via del Vespro 133, 90127 Palermo, Italy; walter.mazzucco@unipa.it

**Keywords:** nutrition, eHealth, wearable technology, digital health, mobile apps, behavior change, prevention, aging, umbrella review

## Abstract

**Background/Objectives:** The increasing use of electronic devices is reshaping lifestyle by offering new avenues for health behavior change. These tools provide to monitor health, fitness, and nutrition, promoting healthier lifestyles to prevent non-communicable diseases (NCDs). This umbrella review (conducted according to PRISMA 2020 guidelines, registered on PROSPERO CRD42024511141) assesses the effectiveness of wearable devices and mobile applications in improving healthy lifestyle behaviors to mitigate the risk of NCDs. **Methods**: Systematic reviews and meta-analyses (*n* = 27) focusing on digital tools for health behavior change were analyzed, with emphasis on their integration into daily life and their impact on health outcomes, including body weight, metabolic and anthropometric parameters, and dietary quality. **Results and Conclusions**: Interventions leveraging gamification, social interaction, and goal-setting (6/27) have shown greater efficacy in improving body-nutrition profile. The integration of eHealth technologies holds transformative potential for preventive healthcare and positive biology. These tools can contribute to healthier lifestyles, extended life expectancy, and reduced healthcare costs, although current limitations exist, including data accuracy, privacy concerns, and sustaining user engagement over time.

## 1. Introduction

The adoption of electronic devices is rapidly transforming health management practices worldwide. People increasingly rely on the internet and social media for guidance on medical-health topics, exercise routines, and nutrition. Leveraging the persuasive power of these platforms, alongside advancements in electronic device technologies, presents a unique opportunity to improve lifestyle habits, particularly in the areas of nutrition and physical activity [1].

Smartphones have facilitated the widespread use of wearable devices and mobile applications (apps), enabling individuals to seamlessly integrate healthy practices into their daily routines. By providing instant access to expert advice, personalized recommendations, and real-time feedback, these tools empower users to adopt and maintain healthier lifestyles. In this context, electronic health (eHealth) and its subset mobile health (mHealth) have emerged as pivotal innovations, revolutionizing healthcare delivery through the integration of information technology and telecommunications.

mHealth, through devices such as smartphones, tablets, and wearable technologies, offers unparalleled opportunities for monitoring individual health metrics and preventing non-communicable diseases (NCDs). These conditions, including cardiovascular diseases and diabetes, are heavily influenced by modifiable risk factors such as sedentary behavior, poor diet, and obesity. Wearable devices and apps enable the tracking of these factors, facilitating early intervention and contributing to increased life expectancy [2].

In Western countries, where rising obesity rates and aging populations exacerbate the burden of NCDs, the need for innovative prevention strategies is urgent. Epidemiological evidence highlights a strong link between elevated body mass index (BMI) and the risk of developing several cancers [3]. By 2050, an estimated 33.6% of the Italian population will be over the age of 65, further emphasizing the need for proactive health interventions to mitigate age-related cardiovascular and cerebrovascular risks [4].

The functionality of mHealth technologies extends beyond simple data collection to include tracking health trends, offering personalized lifestyle recommendations, and addressing behaviors such as poor nutrition and insufficient physical activity. These tools hold the potential to prevent diseases, promote well-being, and reduce the economic burden on healthcare systems by shifting the focus from treatment to prevention [5]. In particular, apps and devices designed to monitor caloric intake, encourage hydration, and promote the consumption of fruits, vegetables, and healthy fats exemplify the role of mHealth in fostering healthier habits [6].

Wearable technologies, including smartwatches and bracelets, have further enhanced personal self-awareness by enabling the tracking of metrics such as physical activity, sleep patterns, and dietary habits. Notably, wearable cameras have shown promise in increasing self-awareness among individuals with chronic illnesses or neurological disorders by capturing data on food intake, sedentary behavior, and physical activity [7,8].

This umbrella review is conducted within the framework of the DARE (DigitAl lifelong pRevEntion) project, which seeks to develop advanced digital methodologies for public health improvement. By focusing on forecasting, surveillance, and prevention, the project aims to create innovative eHealth and mHealth tools to reduce unhealthy lifestyles and prevent the onset of NCDs.

The primary research question of this review is to evaluate the effectiveness of wearable devices and mobile applications in supporting healthy lifestyle behaviors and nutritional management. Specifically, the review aims to systematically synthesize current evidence, identify the main outcomes achieved, and assess the potential of these digital interventions for preventing non-communicable diseases and promoting overall health.

## 2. Materials and Methods

### 2.1. Search Strategy

The search strategy used followed the umbrella review methodologies. Umbrella reviews involve a systematic search and selection of literature to map out key concepts and summarize a range of evidence aimed at conveying the breadth and depth of a particular research field. The choice of an umbrella review was weighted on the basis of the heterogeneity of the results collected—depending on the tool used and the population interested—to define how mHealth can help in nutritional management [9].

The present review followed the PRISMA 2020 checklist (Preferred Reporting Items for Systematic Reviews and Meta-Analyses) [10]. This study has been registered on PROSPERO version 1.1 (https://www.crd.york.ac.uk/PROSPERO/, accessed on 19 February 2024), with registration number CRD42024511141.

A search for studies published between 2013 and 2024 was conducted in the period between December 2023 to March 2024 via three databases: PubMed Medline, Scopus, and EBSCO. The year 2013 was chosen on the basis of bibliometric analysis, indicating that relevant evidence related to e- and mHealth focused on nutrition was not published before this year [11]. The bibliographic search included applying filters to narrow the investigation to systematic reviews, and meta-analyses to have a significant and unique result on how technological tools can have a role in nutritional management, focusing on a population aged 19–64 years which is the main age range able to use the new technology without support and allowed to buy or download mobile apps. The search language was limited to English.

A comprehensive and synthetic search strategy was developed on the basis of keywords found in the titles and abstracts of relevant articles. These included a combination of terms related to the type of digital tool on which the analysis of the paper is focused (digital device, app, wearable/portable/mobile device, sensor, smartphone, smartwatch, phone, eHealth, mHealth), key outcomes related (food intake, food behavior, diet, nutrition), and the aim of the tools used (effectiveness, monitoring, prevention) to better manage the nutritional status of subjects involved.

A second phase of the research aimed to evaluate the effectiveness and utility of digital tools used in the primary studies extrapolated from the systematic reviews included: a focus on app name and platform where to download them, type of wearable device, purpose, intervention period and major outcome indices to understand which one best suited to everyone’s needs. Therefore, a search was conducted for apps and another for wearables via the same filters, followed by a manual search of the references of selected eligible records.

Title, abstract, and keywords of identified papers were screened in duplicate by a pair of independent reviewers. Full-text screening was also conducted by multiple pairs of independent reviewers, and reasons for exclusions were recorded. Other reviewers were consulted to resolve conflicts at both phases of study selection.

### 2.2. Eligibility Criteria

Systematic reviews and meta-analyses of (quasi)experimental studies (i.e., randomized control trials (RCTs), prepost designs, and pilot studies were included in this umbrella review, which focuses on healthy individuals or those with dysmetabolic conditions predisposing them to age-related diseases (e.g., overweight/obesity). The majority of the papers selected lay in the range from 18 to 64 years old. However, few papers described a population above or under the selected range. Reviews targeting overweight/obese individuals without disabling chronic conditions or other diagnosed diseases were retained. Reviews eligible for inclusion reported interventions using digital technology to assess the nutritional status of subjects and related metabolic/anthropometric parameters (e.g., body weight, body fat, waist circumference, biochemical markers, dietary intake, energy intake and expenditure) or to evaluate changes in eating behavior as primary or secondary outcomes. The digital technology encompassed the use of mobile devices (e.g., smartphones, tablets, and PCs), wearable devices (e.g., accelerometers, pedometers, sensors, and portable cameras), and mobile applications (e.g., stand-alone apps or those associated with wearable devices). Additional selection criteria included a focus on behavioral interventions aimed at improving unhealthy lifestyle (diet, physical activity) and/or preventing overweight and obesity. Reviews focusing on apps or wearable devices for monitoring parameters during pregnancy, as well as those exclusively including children or elderly populations, were excluded. Additionally, reviews that did not report results related to subjects’ nutrition or diet, that focused solely on wearable devices reporting physical activity as a primary or secondary outcome, or that addressed secondary or tertiary prevention interventions, were also excluded. Systematic reviews that exclusively included children or ≥65 years old populations were also excluded although we included two papers evaluating the effect of the digital tools on the parents’ food choice.

### 2.3. Data Extraction and Critical Appraisal

Data extraction was conducted independently by one reviewer and verified by a second reviewer. Specific data were extracted regarding review characteristics (e.g., type of e- and mHealth devices, aim of the app/wearable device, type of intervention, health behavior targeted), inclusion criteria based on Participants, Interventions, Comparators, and Outcomes (PICO) process, and results (e.g., key findings of the included studies).

A critical appraisal of all included reviews was performed using AMSTAR 2 (A MeaSurement Tool to Assess systematic Reviews), a tool designed for assessing systematic reviews that include randomized or nonrandomized studies of healthcare interventions [12]. The AMSTAR checklist evaluates various criteria, each representing specific aspects of conducting a systematic review. AMSTAR 2 assesses review quality across critical domains such as protocol registration, adequacy of literature search, reasons for exclusions, risk of bias in included studies, adequacy of meta-analytic methods (if applicable), risk of interpretative bias, and assessment of publication bias.

According to AMSTAR 2 guidelines, reviews meeting all (7/7) or all but one (6/7) critical domains were considered “high” or “moderate” quality, respectively. Reviews meeting all but one critical domain and showing minimal deficiencies in non-critical domains were classified as “low” quality, while those failing to meet multiple critical domains were categorized as “critically low” [13].

## 3. Results

**Figure 1:**
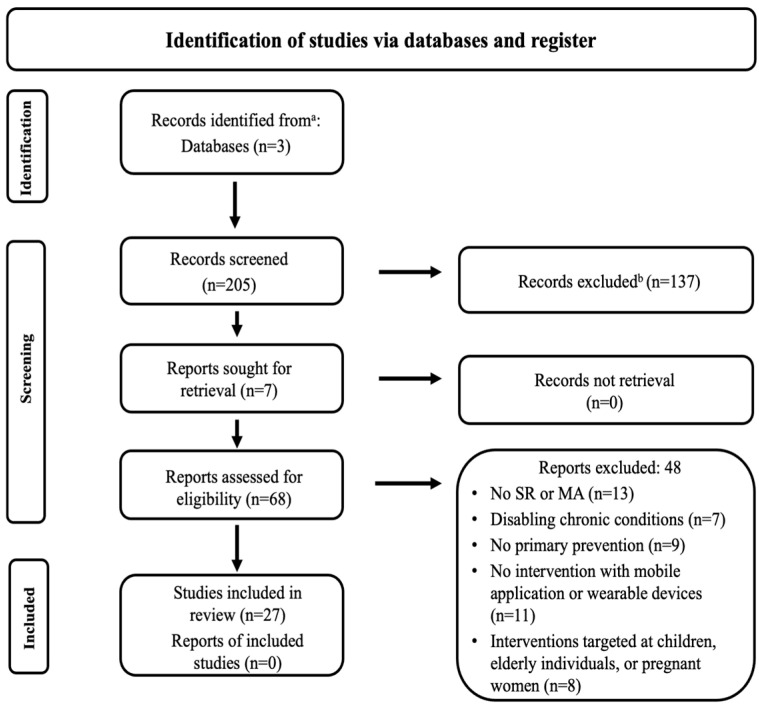
Flow diagram of the study selection process. Abbreviations: SR: systematic review; MA: meta-analysis. ^a^: consider, if feasible to do so, reporting the number of records identified from each database or register searched (rather than the total number across all databases/registers). ^b^: if automation tools were used, indicate how many records were excluded by a human and how many were excluded by automation tools.

A flow diagram illustrating the study selection process is shown in Figure 1.

The PubMed, Scopus, and EBSCO searches yielded a total of 212 records. Specifically, using the keywords listed in the Supplementary Materials, 205 reviews were initially identified. An additional 7 records were obtained through manual searches of references within the selected eligible records. Among the 205 reviews, titles and abstracts were screened according to predefined inclusion and exclusion criteria, resulting in the exclusion of 137 records due to duplication or failure to meet eligibility criteria.

Sixty-eight reviews proceeded to full-text review, during which 48 records were further excluded for various reasons: interventions targeted children, elderly individuals, or pregnant women; document types were not systematic reviews or meta-analyses; populations targeted by interventions had disabling chronic conditions; interventions were not for primary prevention; or m-Health interventions did not include the use of apps or wearable devices.

Ultimately, 27 studies were identified as involving the predefined criteria, comprising 20 from the initial keyword searches and an additional 7 from manual research. Consensus on study inclusion was reached among at least 3 authors.

### 3.1. Search Flow Results

The umbrella review includes a total of 27 studies, each categorized and described based on several key attributes, as detailed in Table 1.

### 3.2. Characteristics of the Included Reviews

The data extracted from Table 1 were synthesized to align with the objectives of the umbrella review. Common themes and trends across the studies were identified and narratively described to provide a cohesive overview of the current landscape regarding digital tools for nutritional management.

The included reviews were published between 2015 and 2023, with the majority from 2020 to 2023 (*n* = 18). The studies were conducted in various countries including the United States (*n* = 4), United Kingdom (*n* = 1), Ireland (*n* = 1), Australia (*n* = 7), Spain (*n* = 4), Germany (*n* = 4), and Asia (*n* = 6). All 27 reviews included RCTs. The selected reviews also encompassed pilot studies, short reports, pre-post studies, case–control studies, and quasi-RCTs.

The age range of participants varied across the included reviews, with a predominant focus on populations aged over 18 years (16/27). Six reviews specified exact age ranges for mHealth interventions [7,18,23,26,31,33]. Two reviews included interventions targeting both adults and children [16,30], while four reviews focused on adults and adolescents [6,8,26,30]. Two reviews involved populations under 35 years and over 40 years [18,19,25,26,28,33]. Only two reviews targeted interventions aimed at parents [20,32], while two were focused on the general adult population without specific age limits [14,24].

Five reviews exclusively focused on overweight or obese subjects [15,24,27,31,34], while the remaining reviews examined healthy populations. Although all reviews assessed the effectiveness of e- and mHealth interventions on changes in nutritional status or eating behavior, only 10 out of 27 reported the use of behavioral change techniques (BCT) [6,7,8,16,20,21,24,25,30,32].

The most common types of e- and mHealth interventions included smartphone applications (16/27) and wearable devices (11/27). All reviews included studies using stand-alone applications, except one [34] that involved interventions combining self-monitoring apps with health coaching.

Parallelly, digital components used to deliver the intervention were in most studies a miscellaneous of different tools integrated into the experimental intervention or control groups. These tools included messaging systems, websites, accelerometers or pedometers designed to stimulate processes such as real-time self-monitoring of diet and physical activity, personalized monitoring of progress and the provision of feedback.

In fact, our research showed that, 9/27 reviews incorporated text messages, 9/27 included websites, 9/27 mentioned accelerometers, 7/27 included pedometers either alone or in combination, and 2/27 discussed the use of wearable cameras to monitor lifestyle behaviors.

#### Main Outcomes of the Included Reviews

The primary outcomes assessed in the reviews were generally related to weight, BMI and waist circumference, changes in eating behavior, improved diet quality, changes in HDL-C, LDL-C, HbA1C, glucose and lipid levels, increased physical activity and reduced sedentary behavior.

Specifically, among the 27 selected records, 9 systematic reviews examined smartphone applications’ effectiveness in monitoring various health aspects (anthropometric, metabolic, and dietary outcomes). Seven of these reviews investigated interventions supporting weight loss or health measures related to weight (e.g., BMI, waist circumference) in healthy or overweight and obese populations [14,15,23,24,27,31,34]. These interventions typically included features such as real-time diet and exercise self-monitoring, personalized progress tracking, feedback provision, and integration with smart devices for data synchronization [14,15,23,27,31,34]. Additionally, 5 reviews focused on combining a healthy diet with interventions using wearable devices to increase physical activity levels. When coupled with personalized nutritional plans, increased physical activity effectively reduced mortality and cardiovascular disease risk factors [4,22,26,30,33]. Two reviews explored monitoring sedentary behavior through wearable devices to promote healthier lifestyles [17,18].

For instance, Hall et al. (2020) highlighted that increasing daily steps by as little as 1000 can significantly reduce all-cause mortality and cardiovascular disease morbidity in adults, underscoring the health benefits even below the traditional 10,000 steps per day [22]. Furthermore, Heesch et al. (2018) emphasized the potential of wearable devices in promoting physical activity and mitigating the adverse effects of sedentary behavior, suggesting tailored accelerometers for specific age groups to enhance long-term usability [18].

Two reviews specifically examined eHealth nutritional interventions, including those via mobile apps, to support users in achieving nutrition-related outcomes such as weight reduction or changes in eating behavior.

These interventions included reminder systems, coaching calls, educational content sharing, motivational messages and tools for recording eating behavior and monitoring physical activity [17,19]. One review highlighted protocols for assessing dietary quality in digital weight loss interventions, focusing on digital diet registers linked to food databases for feedback on daily energy intake, but not for improving diet quality or changing eating behaviour [36].

Among the 27 reviews, 10 primarily focused on the effectiveness of mHealth-assisted nutritional interventions in changing or improving nutritional behaviors [6,7,8,16,20,21,26,29,32,33].

Seven of these reviews assessed app-based interventions’ effectiveness in changing health outcomes as a result of improved nutritional behavior in the subjects studied. These results included obesity indices (e.g., body weight, BMI, waist circumference) and blood chemistry parameters associated with food intake (e.g., glucose levels, lipids, cholesterol, vitamin D, calcium, blood pressure) [6,16,21,22,26,29,35]. Most interventions incorporated BCT such as goal setting, feedback monitoring, knowledge shaping, and social support [6,8,18,22,28,33].

One review assessed digital communication strategies’ effectiveness in promoting healthy behaviors, noting moderate to high levels of user engagement despite limited evidence on behavior change effectiveness [32]. Lastly, two reviews evaluated health promotion through various interventions, including apps aimed at improving eating behaviors. For instance, Mandracchia et al. assessed self-monitoring mHealth apps to increase fruit and vegetable intake, while Zarnowiecki et al. examined nutritional promotion through websites and apps targeting parents’ positive influence on children’s nutrition [7,20].

In summary, we found that twenty-one reviews evaluated digital technologies as tools for health promotion [4,6,7,14,15,16,19,22,27,29,31,32,33,34] and disease prevention [8,17,24,25,26,29,33]. See Table 1 for an overview.

### 3.3. Methodological Quality

The AMSTAR 2 tool was used to evaluate the methodological quality of the systematic reviews and meta-analyses, see Table 2 for the quality rating associated with each paper.

AMSTAR 2 Quality Rating: High (no or one non-critical weakness); Moderate (more than one non-critical weakness); Low (one critical flaw with or without non-critical weaknesses); Critically low (more than one critical flaw with or without non-critical weaknesses).

**Figure 2:**
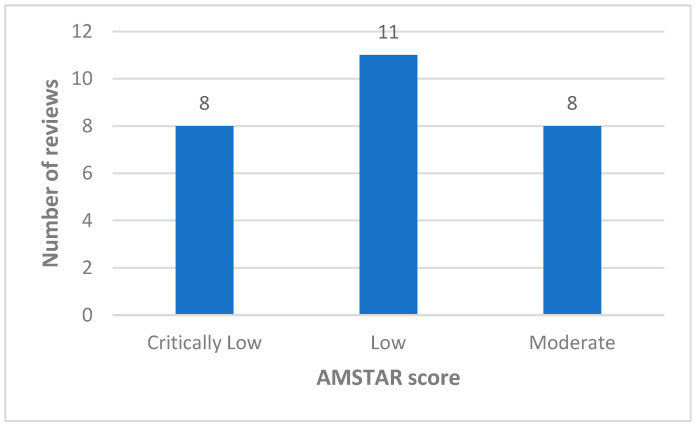
Quality appraisal of the included systematic reviews according to the AMSTAR 2 checklist. Abbreviations: AMSTAR 2: A MeaSurement Tool to Assess systematic Reviews.

AMSTAR 2 tool consists of 16 items including 7 critical domains influencing the overall quality rating. Less than half of the included reviews (11/27) were rated as low quality [7,8,15,18,19,23,25,26,27,29,32], while 8 reviews were assessed as moderate quality [6,14,24,28,30,31,34,35], and 8 reviews were critically low quality [4,16,17,20,21,22,33,36]. A visual summary of the quality appraisal results is provided in Figure 2.

For the critical domains, a significant majority of reviews provided a list of excluded studies [19/27]. Fourteen reviews used a partially appropriate method to analyze the risk of bias, while 11 reviews did not conduct this analysis. Twenty reviews adequately described their review methods, and almost all reviews reported a sufficiently comprehensive search strategy (25/27) or an adequate one (1/27). Nine reviews employed appropriate methods for statistical combination of results, with one review using this method partially. Most reviews considered the risk of bias in individual studies to interpret results (19/27), while 8 reviews did not assess this aspect. Only 9 reviews adequately explored publication bias and its potential impact on review outcomes.

In terms of non-critical domains, 25 reviews incorporated PICO components and conducted a systematic literature search. All reviews, except one, described study selection and data extraction processes performed independently by at least two authors, and disclosed potential conflicts of interest.

A significant proportion of authors addressed result heterogeneity within their reviews (21/27), and about one-third assessed the potential impact of bias risk on meta-analysis results (10/27). Few reviews provided detailed descriptions of included studies (3/27) or disclosed funding sources (7/27). For detailed criteria, refer to Table 3.

### 3.4. App and Wearable Device-Based Health Promotion Interventions

M-Health leverages information technology and mobile communication tools such as mobile phones, tablets, and wearable devices to facilitate public health initiatives. These technologies offer convenient and personalized methods for communication, education, sharing, and health monitoring, often through apps either alone or in conjunction with wearable devices. The evolution of smartphones equipped with apps, internet connectivity, cameras, accelerometers, and video technology has significantly enhanced accessibility and interactivity in app-based interventions [4,8,17,18,22,24,26,27,28,30,33,34,35].

To achieve this, we conducted a detailed analysis of the systematic reviews and meta-analyses included in our umbrella review to identify primary studies that used mobile apps and wearable devices to assess or monitor the nutritional status of the population or to monitor changes in dietary behavior. We specifically reviewed interventions validated in the literature that utilize mobile apps and wearable devices. Based on this analysis, we evaluated the effectiveness and utility of interventions involving mobile apps and wearables separately, considering standalone app interventions as well as combinations with other tools.

#### 3.4.1. App-Based Interventions and Their Outcomes

Table 4 presents the characteristics of mobile app intervention studies identified in the systematic reviews.

Apps were utilized for monitoring individual health status (10 out of 28), behavior modification (14 out of 28), or a combination of both (4 out of 28), offering feedback on personal health and delivering health-related information (16 out of 28). Following mobile app intervention, significant improvements were observed in the primary outcomes of the studies.

Most RCTs included a two-arm intervention design consisting of an intervention group and a control group. Intervention groups were provided a mobile app and additional interventions such as SMS, phone calls, mail, website, paper diaries, and pedometers (9 out of 12). Additionally, in 7 out of 28 studies, control groups received no intervention, while the remainder were provided with either a mobile app or website, diary, and pedometer.

Interventions showing significant improvements in both behavioral and health outcomes typically involved single and multicomponent app interventions. These interventions had sample sizes ranging from 34 to 566 participants and durations longer than 4 week [37,38,39,40,41,42,43,44,45,46,47,48]. Most outcomes were observed in the short-term (up to 8 weeks) and medium-term (up to 12 months), with fewer studies reporting long-term outcomes beyond 12 months, up to 24 months [43,44,49].

A primary finding was to identify studies that measure mobile apps and their impact on weight loss and diet quality of the participants. In total, 8 studies implemented and described app interventions intended to improve anthropometric, metabolic, and dietary outcomes with significant results.

Eisenhauer et al. (2021) assessed the use of a popular smartphone app (LoseIt!) for dietary self-monitoring and weight loss by comparing it with traditional diet counseling and entry methods [48]. App users recorded dietary data more consistently compared with other groups and lost weight after 8-weeks (95% CI: 2.22, 6.06) [48]. In Balk-Moller et al. (2017) study, an application (SoSu-life) developed to assist in weight reduction among specific employee groups within workplace settings [44]. The SoSu-life app was also employed to aid participants in achieving their personal goals and to provide “challenges among colleagues” and “weekly challenges” to engage and compete with other groups. At 38 weeks, the SoSu-life group had a larger decrease in body weight (−1.01 kg, *p* = 0.03), body fat percentage (−0.8%, *p* = 0.03), and waist circumference (−1.8 cm, *p* = 0.007) compared with the control group [44].

In the Social Mobile Approaches to Reduce weighT (SMART) study, Godino et al. (2016) assessed the efficacy of a two-year social and mobile intervention designed to reduce weight by improving weight-related behaviors among college students [43]. The SMART intervention was the first to incorporate several theory-based behavior change techniques previously demonstrated to be effective in improving weight-related behaviors. Although, within the intervention group, the use of apps (GoalGetter App, BeHealthy App, TrendSetter App) facilitated moderate short-term weight loss at 6 (−1.33 kg, *p* = 0.011) and 12 months (−1.33 kg, *p* = 0.008), it did not result in weight reductions at 18 and 24 months (−0.67 kg, *p* = 0.200) [43].

Hales et al. (2016) in a randomized clinical trial highlighted significant weight loss in the intervention group (−5.3 kg, CI: −7.5, −3.0) that used a behavioral self-monitoring weight loss app (Social POD App) aimed at social support and self-monitoring of diet, physical activity and weight, compared to commercially available tracking app (−2.23 kg, CI: −3.6, −1.0) [41]. In Zhou, et. al., (2016), the primary objective was to assess the impact of the smartphone-based diabetes management application, (Welltang), on glycated hemoglobin (HbA1c), while the secondary objective was to measure whether Welltang improved blood glucose, low-density lipoprotein cholesterol (LDL), weight, and self-care behaviors [42]. This study has shown that diabetes patients using the Welltang application achieved statistically significant improvements in HbA1c, blood glucose, however, there were no significant variations observed in LDL levels and weight [42]. Instead, Bentley et al. (2016) focused on improving the quality of the participant’s diet by using the app AiperMotion 500 supported by feedback and focus groups [50]. In this study, both weight loss and Hba1c levels showed a more significant reduction compared to the control group [50].

Other studies have also investigated the efficacy and utility of app-based interventions, but no significant variations were found about weight loss, anthropometric parameters, and dietary quality [51,52,53,54,55,56].

In total, 7 studies have investigated the connection between the use of mobile technology and the improvement of dietary quality or eating behavior, showing positive results [38,40,45,47,57,58,59].

Gilliland et al. (2015), have described the “SmartAPPetite” research project: a smartphone application, designed to encourage healthy eating by reducing educational, behavioral, and economic barriers to accessing healthy, local food [40]. One of the most noteworthy findings is that involvement with SmartAPPetite had a direct effect on consumption of healthy foods [40].

In the pilot study by Ipjian et al. (2017), it was examined how the impact of smartphone technology could facilitate dietary change in healthy adults, with the main focus on diet quality and its effect on sodium intake [59]. Although diet quality scores were comparable with control groups, baseline values for urinary sodium excretion decreased in mobile app users My FitnessPal App (−838 ± 1093 mg/24 h) compared to control group (+236 ± 1333 mg/24 h), respectively (*p* = 0.010) [59].

In order to promote healthy eating behaviors, 3 studies have tested the effectiveness of apps (VeggieBook, Vegethon App) to increase the use of vegetables in meal and snack preparation through goal setting, self-monitoring, and personalized feedback. The results of the studies suggested that the use of these apps might be a feasible way to increase vegetable consumption among adults attempting to lose weight, at least in the short term [45,57,58].

**Table 4:** Features and outcomes of the app-based health promotion interventions analyzed in 28 studies.

Author, Year	Sample Size Total	Experimental Group	Control Group	App Name	Platform	App Purpose	Intervention Period	Major Outcome Indices	Ref.
Lee, W. et al., 2010	36	19	17	SmartDiet	App Store	-Monitor health status and behavior change -Dietary quality -Provide information	6 Weeks	-Fat mass -Body weight -^a^ BMI	[37]
Carter, M.C. et al., 2013	129	43 (App)	86 (Web, paper diaries)	My Meal Mate App	^b^ N/A	-Provide information, feedback -Monitor health status	6 months	Weight loss	[51]
Van Drongelen, A. et al., 2014	502	251 (App, website)	251 (Website)	MORE Energy App	Play store	-Provide information, feedback -Monitor behavior change	6 months	-Snacking behavior -Physical activity -Sleep quality	[38]
Laing, B.Y. et al., 2014	212	105 (App, usual care)	107 (Usual care)	MyFitnessPal App	App store/Play store	-Monitor behavior change	6 months	-Self-monitoring adherence	[52]
Nollen, N.L. et al., 2014	51	26 (app)	25	MyPal A626	Microsoft Store	-Weight control -Monitor behavior change -Provide information, feedback	12 weeks	-Increase fruit and vegetables -Decreasing sugar drink	[60]
Wharton, C.M. et al., 2014	34	19 (mobile app)	15 (paper and pencil)	Lose It!	App Store/Play Store	-Monitor health status and behavior change	8 weeks	-Weight loss	[39]
Gilliland, J. et al., 2015	208	N/A	N/A	SmartAPPetite	Play Store	-Dietary quality -Provide information -Monitor behavior change	10 weeks	-Healthy food consumption	[40]
Hales, S. et al., 2016	51	26 (^c^ TBP + App Social)	25 (TBP + App Standard)	Social POD app	N/A	-Provide information -Monitor health status	12 weeks	-Weight loss -BMI	[41]
Allman-Farinelli, M. et al., 2016	250	125 (App, SMS, call, e-mail)	125 (call, SMS)	TXT2BFiT	N/A	-Dietary quality -Monitor health status and behavior change	12 weeks	-Weight loss -Improvement in eating behavior	[61]
Zhou, W. et al., 2016	100	50 (app)	50 (standard of care)	Welltang	N/A	-Reduce HbA1c level -Improve diabetes status -Monitor health status	3 months	-Improvements in HbA1c -Improvements in glycemia -Knowledge of diabetes and self-care behaviors	[42]
Bentley, C.L. et al., 2016	27	9 (app)	18 (no app)	AiperMotion	N/A	-Reduce HbA1c level	12 weeks	-Reduce HbA1c levels -Weight loss	[50]
Godino, J.G. et al., 2016	404	202 (Mobile app, facebook, text messaging, emails, website)	202 (Website, emails)	GoalGetter App, BeHealthy App TrendSetter App	App Store/Play Store	-Provide information, feedback -Monitor Health Behavior	12 months	-Body weight, -BMI, -Waist circumference -Blood pressure	[43]
Elbert S.P. et al., 2016	146	146	N/A	Fruit and Vegetables hAPP	Play store	-Monitor behavior change -Dietary quality -Predict fruit and vegetable intake	6 months	-Intake of fruits and vegetables	[62]
Mummah, S.A. et al., 2016	17	8	9	Vegethon App	N/A	-Provide information, feedback -Monitor behavior change -Increase vegetable consumption	12 weeks	-Daily vegetable consumption	[57]
Martin, C.K. et al., 2017	40	20 (smartphone)	20 (usual care)	SmartLoss	N/A	-Provide information -Dietary quality -Monitor health status	16 weeks	-Weight loss	[53]
Balk-Møller, N.C. et al., 2017	566	355 (app)	211 (no app)	Sosu-life	N/A	-Monitor health status	38 weeks	-Weight loss -Body fat -Waist circumference	[44]
Spring, B. et al., 2017	96	96 (Self-guided, standard or technology-supported)	N/A	ENGAGED	N/A	-Provide information, feedback -Monitor health status	6 months	-Body weight -Self-monitoring adherence	[54]
Mummah, S. et al., 2017	135	68	67	Vegethon App	N/A	-Provide information, feedback -Monitor behavior change	8 weeks	-Increased intake of vegetables	[58]
Hull, P. et al., 2017	80	80 (App)	N/A	CHEW App	N/A	-Nutrition education	3 months	-Dietary quality -Healthy snacks and beverages intake	[63]
Ipjian M.L. et al., 2017	30	15 (App, website, dietary sodium intake, verbal instruction)	15 (Journal, dietary sodium intake, verbal instruction)	MyFitnessPal App	App store/Play store	-Provide information, feedback -Monitor behavior change	4 weeks	-Urinary sodium excretion	[59]
Clarke, P. et al., 2019	300	189	111	VeggieBook	App Store	-Dietary quality -Monitor behavior change	10 weeks	-Increased use of different types of vegetables	[45]
Ambrosini, G.L. et al., 2018	50	50	N/A	Easy Diet Diary App	App store	-Monitor behavior change	1 Week	-Energy supply -Sugar intake	[64]
Delisle Nyström, C. et al., 2018	263	133 (App, push notifications, graphical feedback)	130 (Pamphlet)	MINISTOP App	App store/Play store	-Provide information, feedback -Monitor health status	12 months	-BMI -Fruits and vegetables intake -Reduced candy, and sweetened beverages	[55]
Haas, K. et al., 2019	43	43 (app)	N/A	Ovivia App	App Store/Play Store	-Dietary quality -Provide information -Monitor health status and behavior change	6 months	-Weight loss -BMI -Waist circumference -Body fat -Blood pressure -Eating habits	[46]
Patel, M.L. et al., 2019	105	105 (App, e-mail)	N/A	MyFitnessPal	App store/Play store	-Provide information, feedback -Monitor health status	12 weeks	-Weight loss -Self-monitoring adherence	[56]
Eyles, H. et al., 2017	66	33 (app)	33 (usual care)	SaltSwitch	N/A	-Dietary quality -Monitor behavior change	4 weeks	-Significant reduction in purchases of salty foods	[47]
Rosas, L.G. et al., 2020	191	92 (App, website, usual care, activity tracker)	99 (Usual care, activity tracker)	MyFitnessPal	App store/Play store	-Provide information, feedback -Monitor health status	24 months	-Body weight -Waist circumference -Psychosocial well-being	[49]
Eisenhauer, C.M. et al., 2021	80	40 (mobile plus)	40 (mobile basic)	Lose-It!	App Store/Play Store	-Dietary quality -Monitor health status	6 months	-Weight loss	[48]

Abbreviations: ^a^ BMI, Body Mass Index; ^b^ N/A, not applicable; ^c^ TBP, theory-based podcast.

In Eyles et al. (2017), the main aim of the study was to determine the effectiveness of SaltSwitch, an innovative smartphone application (app) that enables shoppers to scan the barcode of a packaged food and receive an immediate traffic light nutrition label on the screen, to support people with to make lower salt food choices [47].

Ambrosini et al. (2018) tested the feasibility of using a smartphone app (Easy Diet Diary App) for use as an epidemiological dietary assessment tool, compared with a traditional dietary assessment method [64]. The quality of the participant’s diet was measured by average energy ratios from macronutrient fiber, iron, and calcium densities from the app and the 24 h recalls. The study indicated no distinct differences between the app and the 24 h recalls for protein, saturated fat, carbohydrate and iron density. However, added sugar intake was higher in those in the 24 h recalls [64].

Finally, 3 studies assessed apps and their influence on weight loss, consequently leading to an enhancement in participants’ dietary quality [39,46,61].

Wharton et al. (2014) assessed the use of a popular smartphone app (Lose It! App) for dietary self-monitoring and weight loss by comparing it with traditional diet counseling and entry methods [39]. Their hypothesis posited that participants using the smartphone app for data recording would demonstrate lower attrition rates in the study and achieve greater weight loss. The study showed that weight loss was similar across all groups [39].

In a RCT by Allman-Farinelli et al. (2016), was examined a 12-week m-Health program (TXT2BFiT) on prevention of weight gain in young adults and lifestyle behaviors [61]. This included coaching calls, text messages, emails, apps, and downloadable resources from the study website. Lifestyle behaviors addressed were intake of fruits, vegetables, sugar-sweetened beverages (SSBs), take-out meals, and physical activity. Delivery of this mHealth intervention resulted in modest weight loss at 12 weeks compared with controls (95% CI −6.1, −1.3) and after 9 months (95% CI −6.9, −1.8). Although there was no evidence of change in physical activity, improvements in dietary behaviors occurred [61].

Additionally, in another study by Haas et al. (2019), the effectiveness and feasibility of weight loss counseling by dietitians using a mobile phone app (Oviva App) for patients with overweight and obesity was evaluated [46]. The outcomes examined were body weight, fasting glucose, fasting insulin, triglyceride, high-density lipoprotein cholesterol, blood pressure (BP), BMI, waist circumference, body fat, dietary assessment, and health-related quality of life. Changes in anthropometric and metabolic parameters were significant. Furthermore, significant changes in certain eating habits such as higher frequency of vegetable, fruit, and breakfast consumption and lower frequency of alcohol, sweet, and fat consumption were also demonstrated [46].

#### 3.4.2. Wearable-Based Interventions and Their Outcomes

The characteristics of wearable devices analyzed are presented in Table 5.

We achieved our objective through five studies aimed at describing intervention using wearable devices to begin, monitor and maintain individual healthy lifestyle status, with a focus on promoting a combination of healthy diet with physical activity for NCDs prevention [65,66,67,68,69]. Wearable devices were utilized for monitoring individual health status, food intake behavior and its modification to follow a healthy lifestyle.

Two reviews, in particular, focus their studies on wearable cameras thanks to their high power of usability and affordable costs [65,66]. Wearable cameras are used especially for dietary assessment.

The discrepancy between the self-reporting and the daily energy intake makes it necessary to oversee subjects following a food plan. Gemming L. et al. (2015), in fact, confirm that the wearable camera “SenseCam” is worthwhile to accurately monitor what people actually eat every day [66]. Wearable cameras make the difference as the report of what has been eaten is not bound by what the subject remembers and furthermore, compared to the method of doubly labelled water usually used, it is much more accurate thanks to the video or picture recording within 24 h.

Doherty A.R. et al. (2013) analyzed the advantages of the wearable camera of Microsoft SenseCam to monitor food intake, nutrition-related behaviors, and also physical activity [65].

Monitoring dietary habits based on self-reporting can sometimes not be truthful especially regarding portion sizes and the tendency to consume excessive snacks outside of scheduled meals.

As adhering to a structured dietary plan is challenging, subjects who used the SenseCam confirm that the major benefit is that the self-reporting error about food eaten and sedentary behaviors is minimized thanks to the capture of images every 20 s. Indeed, it is noticed that for those who wear the wearable camera it is easier to remember the food consumed and associate it with the energy intake. The only disadvantages are represented by the low resolution of the pictures and violation of privacy regarding daily activities.

Not only wearable cameras but also innovative wearable devices such as microphones, micro electro-mechanical gyroscopes, motion sensors, electrical and electromyographic sensors have the power to evaluate food intake and healthy lifestyle. McClung et al. (2018) introduced wearable devices able to control food intake by recording the hand-to-mouth gestures (using the InertiaCube3 sensor manufactured by InterSense), detecting food crushing through microphones (in particular FG-23329-CO5 of Knowles Acoustics), swallowing frequency to make the difference between solid and liquid food, or register muscle activations which is correlated to the chewing motion [67]. These innovative devices have been studied to demonstrate that last generation sensors are considerably helpful to monitor the dietary intake by detecting swallowing, especially in community-dwelling individuals. Indeed, this last factor is helpful since it corresponds to the amount of food ingested simply using a pair of smart eyeglasses that accommodate processing electronics and electromyography electrodes.

Yujie Dong et al. (2012) talk about a micro electro-mechanical gyroscope used as a “bite counter” device [68]. The tool, named InertiaCube3, was born to be worn in every moment and everywhere, especially before the meal to track the movement while the subject is eating, turning it on before eating and turning off immediately afterwards. It is very helpful to monitor subjects who have to maintain a correct nutritional plan, especially obese subjects [68]. From the total of the three experiments performed in the paper, in which the focus is on the bites and food intake, it is clear that the device is reliable in terms of monitoring kilocalories consumed daily and associated with bites per meal.

The annoyance caused by noisy chewing can actually be leveraged for practical purposes, such as monitoring chewing patterns. In 2012, Sebastian Päßler and colleagues explored the use of a microphone to count the number of times a person chews [69]. The microphone used are two (FG-23329-CO5 of Knowles Acoustics) sensing when the subject is eating. One microphone is localized into the outer ear canal to register the emitted sounds from the skull bones, the other one is located behind the ear to differentiate noises of the subject from the ones from the environment. The different sounds will identify different kinds of food, rather than quantify food intake, considering that chewing solid food generates more noises compared to liquid like sweet creams or pudding.

This innovative approach gives the opportunity to the medical specialists to better follow patients with eating disorders. Using two microphones, in fact, nuisances can be recorded and associated with specifical foods. Furthermore, these findings lay the groundwork for the development of a broader food list and turn into useful data collection methods in scientific research.

**Table 5:** Features and outcomes of the wearable device-based health promotion interventions analyzed in 5 studies.

Author, Year	Sample Size Total	Experimental Group	Control Group	App Name	Platform	App Purpose	Intervention Period	Major outcome Indices	Ref.
Dong, Y. et al., 2012	102	^a^ N/A	N/A	-Gyroscope	-InertiaCube3 by InterSense	-Record the hand-to-mouth gestures	24 h	-Capture chewing motion -Monitor the number of daily meals	[68]
Päßler, S. et al., 2012	51	N/A	N/A	-Microphone	-FG-23329-CO5 by Knowles Acoustics	-Monitor the food intake of subjects in therapy -Differentiate kinds of food consumed	24 h	-Register chewing noises	[69]
Doherty, A.R. et al., 2013	N/A	N/A	N/A	-Wearable camera	-Microsoft SenseCam	-Measure sedentary behaviour -Monitor nutrition-related behaviours	24 h	-Monitor food intake -Monitor physical activity	[65]
Gemming, L. et al., 2015	40	N/A	N/A	-Wearable camera	-Microsoft SenseCam	-Assess kilocalories consumed (energy intake) -Monitor health behaviours	15 days	-Take pictures of daily meals -Measure of energy intake	[66]
McClung, H.L. et al., 2018	N/A	N/A	N/A	-Microphones -Smart eyeglasses with electromyography electrodes -Motion sensors -Wearable biosensors	-InertiaCube3 -FG-23329-CO5 by Knowles Acoustics	-Assess food intake -Monitor subjects following a healthy nutritional plan -Make the difference between solid and liquid food	24 h	-Detect food crushing -Register swallowing frequency -Register muscle activations -Record hand-to-mouth gestures	[67]

Abbreviations: ^a^ N/A, not applicable.

**Figure 3:**
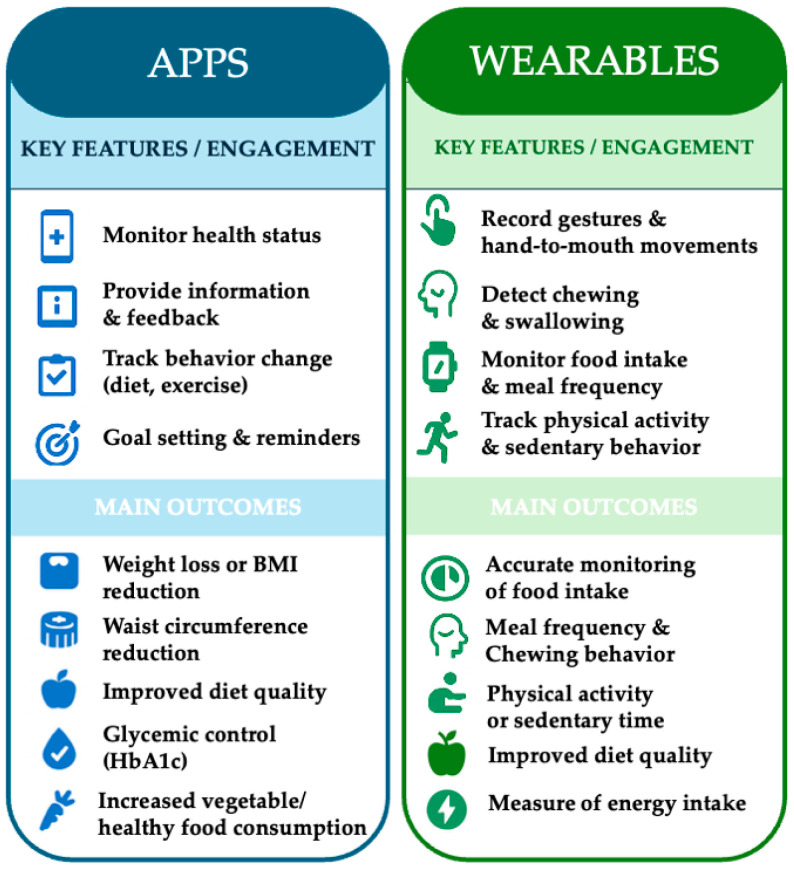
Comparative overview of Apps and Wearables: effectiveness outcomes and engagement features.

Researchers can gather accurate information about eating behaviors in order to monitor health monitoring and even develop technologies for assisting people with eating disorders [69]. A comparative summary of the main engagement features and effectiveness outcomes reported for mobile applications and wearable devices is presented in Figure 3.

## 4. Discussion

The integration of eHealth and mHealth technologies represents a promising frontier for improving nutritional management and preventing non-communicable diseases (NCDs). For instance, the World Health Organization estimates that over 70% of global deaths are attributed to NCDs annually, highlighting the critical role of innovative tools like mobile health apps and wearable devices in addressing this growing health burden. This umbrella review provides an overview of systematic reviews and meta-analyses that evaluate the effectiveness of apps and wearable devices in supporting nutritional management and promoting long-term health outcomes.

The mobile apps analyzed have proven useful in monitoring health status, facilitating behavioral change, and providing feedback and health-related information. Specific applications have been tailored to monitor blood glucose levels in diabetic patients, manage symptoms and provide real-time feedback in oncologic patients, and support management in patients with bipolar disorder [70,71,72]. Moreover, the inclusion of social interaction, team collaboration, and competitive elements between teams has been highlighted as enhancing the effectiveness of mobile app interventions [41,42,43,44,48,50]. Gamification within apps may also improve engagement and knowledge retention among users [73,74,75]. By incorporating elements such as rewards, progress tracking, and interactive challenges, gamification leverages intrinsic motivation and creates a sense of achievement, which are key to sustaining user interest. For example, leaderboards and point-based systems encourage competition and social interaction, further enhancing engagement. These mechanisms foster long-term adherence to health behaviors, particularly when combined with personalized feedback and goal-setting.

Among the selected papers, the apps and wearables that showed the greatest effectiveness on weight loss and/or metabolic parameters shared several key features, including personalized feedback, goal-setting mechanisms, and gamification elements. Apps like the Social POD App and SoSu-life demonstrated the importance of social interaction and user engagement, while devices like AiperMotion emphasized the integration of activity tracking with motivational feedback. For example, the Social POD app provided theory-based notifications and messages to encourage self-monitoring. Participants in the experimental group lost significantly more weight and had greater reductions in BMI than those in the comparison group, with a dropout rate of only 12%. Similarly, the SoSu-life application, through “peer challenges” and “weekly challenges” to engage and compete with other groups, helped participants to achieve their personal goals; indeed, at 38 weeks, the SoSu-life group had a greater reduction in body weight, body fat, and waist circumference than the control group, again without an excessive drop-out rate.

A subset of papers focused on mHealth-assisted interventions for diet quality improvement and monitoring dietary behavior and related health [6,7,8,16,20,21,22,26,28,29,32,33,35]. Positive outcomes include increased fruit and vegetable intake, reduced sodium consumption, and improved dietary choices [38,40,45,47,57,58,59]. SmartAPPetite, for instance, demonstrated effectiveness by delivering food messaging through direct ‘push notifications’ that increased healthy food awareness and consumption [40]. Another app, Vegethon, incorporated “process motivators” such as fun, surprise, and competition, yielding higher daily vegetable consumption in intervention groups compared to controls [57,58]. Notably, further studies are needed to evaluate whether these effects can be sustained over the long term, which is critical for addressing chronic dietary behaviors effectively.

Regarding wearable devices, tools like pedometers and advanced sensors demonstrated positive impacts in promoting physical activity and enhancing self-monitoring. Innovative devices, such as smart glasses with electromyographic electrodes, showed promising potential for monitoring eating behaviors and ensuring adherence to nutritional plans. Despite these advances, challenges such as data accuracy, privacy concerns, and user engagement persist. Moreover, the effectiveness of wearable devices relies heavily on consistent usage; intermittent use does not yield beneficial effects. For example, studies on devices like ActiGraph, Fitbit, and Apple Watch 2 confirm their reliability in monitoring user activities continuously when worn 24/7 [35].

While the reviewed studies demonstrate the potential of eHealth and mHealth technologies in improving nutritional management, several considerations are critical for future implementation. Sustained long-term engagement, adherence, and scalability remain challenging, limiting the generalizability of results. Ethical and privacy issues related to the collection, storage, and use of sensitive personal health data must be addressed. Furthermore, future integration with artificial intelligence and big data approaches could enhance personalization, predictive capabilities, and intervention optimization.

### Limitations

Several limitations should be considered when interpreting the findings of the reviewed studies. Short duration of studies, lack of theoretical foundations, and the predominance of evidence from Western populations hinder accurate estimation of effect sizes and broader applicability. These limitations make it difficult to generalize results to other populations or predict long-term outcomes. Furthermore, heterogeneity in intervention types, outcome measures, and study designs, alongside challenges related to user engagement, adherence, and privacy, further restricts interpretation and scalability of the results. Addressing these methodological and contextual limitations will be crucial to improve the robustness, equity, and translational potential of future digital health research.

## 5. Conclusions

The shift towards digital technologies in healthcare and wellness represents a pivotal advancement in preventive medicine and positive biology. These innovations provide accessible tools for health education and behavior modification, enabling individuals to take proactive control of their health on a daily basis.

To fully realize their potential, future research must address challenges related to data accuracy, user privacy, and long-term adherence through collaborative and theory-driven approaches.

From a practical standpoint, researchers should focus on long-term, methodologically robust studies with standardized outcomes; app developers should integrate validated behavior change techniques and ensure interoperability and ethical data management; and policy makers should promote digital health literacy and equitable access within clear regulatory frameworks.

Furthermore, careful attention should be given to potential unintended consequences of digital tools. For individuals predisposed to disordered eating, continuous monitoring of diet, body weight, or physical activity may exacerbate obsessive or restrictive behaviors, contributing to unhealthy psychological patterns or body image concerns. Similarly, for those facing food or nutrition insecurity, interventions emphasizing dietary optimization may inadvertently highlight resource scarcity or create frustration when access to healthy foods is limited. Designing digital interventions ethically and inclusively is essential to support all users safely and ensure that digital health promotes wellbeing without widening existing disparities.

## Figures and Tables

**Table 1:** Characteristics of the 27 studies included in the Umbrella Review.

First Author, Publication Year	Document Type	Designs of Studies Included in the Review	Country of Author	Age Group (Age Range)—Special Populations	Date Range of the Search	N. of Databases Searched	Overall Review Aim	Outcomes	Type of Tools	Ref.
Mateo, G.F., 2015	SR ^a^, MA ^b^	RCTs ^c^	Spain	Adults (age range not specified)	1960–2015	3	Health promotion, weight loss interventions	Body weight, BMI ^d^, waist circumference	Mobile app	[14]
Semper, H.M., 2016	SR, MA	Quasi-experimental RCTs and RCTs	UK	Adult (>18 ages)	2014 (May)–2015 (April)	16	Health promotion, weight loss interventions	Weight loss	Mobile app	[15]
Schoeppe, S., 2016	SR	RCTs	Australia	Adults and children (age range not specified)	2006–2016	5	Health promotion	Diet, weight loss, BMI, daily fruit and vegetable intake, physical activity and sedentary behavior, blood pressure	Mobile app, messaging tools (text or audio messages), website, pedometer	[16]
Füzéki, E., 2017	SR	Cross-sectional and longitudinal study	Germany	Adults and older adults (≥18 ages)	2007–2016 (March)	4	Summarize available evidence on the relationship between ^e^ LIPA and health outcomes measurable through wearable devices	Obesity, mortality, markers of lipid and glucose metabolism	Wearable motion sensors (accelerometers)	[17]
Heesch, K.C., 2018	SR	Descriptive studies, validation studies, review	Australia	Adults (age ≥60 years)	2017 (December)–2018 (November)	3	Assess the validity and reliability of accelerometers for the assessment of sedentary behavior	Sedentary behavior, older adults	Wearable motion sensors (accelerometers)	[18]
Lee, M., 2018	SR	RCTs	Korea	Adults (<35 years)	1937–2017 (November)	3	Health promotion	Nutrition knowledge, diet quality, body weight, BMI	Mobile App, website, personal coaching, SMS, pedometer	[19]
Maddison, R., 2019	SR	Feasibility, pilot, validation and methodological studies, RCTs	Australia	Adults, school students, older adults (age range not specified)	2010–2017	9	Use of wearable cameras to capture health-related behaviors	Self-management, dietary intake, physical activity, activities of daily living, sedentary behavior	Wearable cameras	[8]
Mandracchia, F., 2019	SR	RCTs	Spain	16–71 years	2008–2018	2	Health promotion	Dietary habits, healthy weight and healthy body fat percentage, ^f^ PA	Mobile App, text and/or audio messages, coaching, emails, accelerometer	[7]
Villinger, K., 2019	SR, MA	RCTs	Germany	Adolescent and adults (age range not specified)	2006–2017	7	Health promotion, nutrition-related health outcomes	Body weight, BMI, clinical parameters (blood lipids)	Mobile app and text messages	[6]
Zarnowiecki, D., 2020	SR	RCTs, cohort and cross-sectional and qualitative studies	Australia	Intervention aimed at parents of children (age range not specified)	2013–2018	5	Nutrition promotion	Healthy food consumption (fruit and vegetable)	Mobile app and website	[20]
Paramastri, R., 2020	SR	RCTs, nested trial, case–control trial, pilot RCT	Taiwan, Pakistan, Qatar, Canada	>18 ages	2010–2018	4	Increase knowledge related to nutrition	Vegetable and sugar-sweetened beverages intake, body composition (fat mass, weight, body mass index), diet behaviors, PA	Mobile apps, coaching calls, text messages, website	[21]
Hall, K.S., 2020	SR	Prospective studies	U.S.A.	Adults (≥18 ages)	1937–2019 (August)	4	Identify daily steps number and evaluate their association with all-cause mortality, ^g^ CVD morbidity or mortality, and dysglycemia	Daily step count	Wearable devices (pedometer and accelerometer)	[22]
Cavero-Redondo, I., 2020	SR, MA	RCTs, non-RCTs, pilot studies	Spain	Adults (20–60 year)	1900–2020	4	Behavioral weight management interventions	Body weight	Mobile App, Website	[23]
McDonough, D.J., 2021	SR, MA	RCT	U.S.A.	Adults (age range not specified)	2019 (December)–2020 (September)	6	Incorporate wearable technologies into physical activity interventions to reduce body weight and BMI	Body weight, BMI, step count	Wearable devices (accelerometer, pedometer)	[24]
Dixit, S., 2021	Review	RCTs, SR, MA	Kingdom of Saudi Arabia	All ages (age range not specified)	2014–2020	3	Explore the possible role of technological advances and social media platforms as an alternative tool in promoting a healthy living style	Physical activity, dietary intervention	Websites, webpages, wikis, mobile devices and apps, social media and social networking channels, video chat, video sharing, podcast media wearable devices, training devices	[4]
Robert, C., 2021	SR, MA	RCTs	Singapore	≥40 years	2014–2019	5	Improve diet and nutrition	Anthropometric measures, clinical outcomes, PA, smoking cessation, medication adherence, behavioral change techniques	Mobile apps, wearable technology, web-based app, phone calls, email, text messages, video-conferencing, tele-health sessions	[25]
Davies, A., 2021	SR	Primary studies, SRs, review	Australia	13–35 years	2008 (January)–2021	7	Validity of new technologies that measure bone-related dietary and physical activity risk factors in adolescents and young adults	Diet and physical activity	Wearable cameras, body-worn monitors, accelerometers online web-based tools, mobile-based tools or apps	[26]
Raber, M., 2021	SR	RCT, experimental and longitudinal	USA	Adults (>20 years)	1937–2020	8	Health promotion (weight loss interventions)	Weight loss	Mobile apps, paper food diaries, wearables, websites and personal digital assistants	[27]
Chevance, G., 2022	SR, MA	SRs, MAs, comparative study, validation study	Spain	Adult (>18 years)	2015 (January)– 2021 (July)	2	Validation of wearable devices as tools used for health outcomes	Heart rate, energy expenditure, step count	Wearable device	[28]
Scarry, A., 2022	SR	RCTs, short report, pilot study	Ireland	Adult (>18 ages)	2010–2020	3	Diet quality improvement	Quality diet, weight loss, diet management, ^h^ HbA1c control, sodium intake, BMI, blood pressure, hemoglobin, ^i^ FPG, and serum lipids	Mobile apps, wearable devices, SMS, email, social networking apps, websites, personal online coaching	[29]
Ferguson, T., 2022	SR	SR, MA	Australia	All ages (age range not specified)	1900–2021 (April)	7	Examine the effectiveness of activity trackers for improving physical activity, physiological and psychosocial outcomes	Step count, energy expenditure, walking, aerobic capacity, ^j^ VO_2_max, psychosocial outcomes	Wearable activity tracker: pedometer, accelerometer, activity monitor, and step-counting smartphone application	[30]
Chew, H.S.J., 2022	SR, MA	RCTs	Singapore	Adults (22–70 years)	1900–2022	7	Health promotion	Weight loss, waist circumference (cm), calorie intake, blood pressure, ^k^ HDL-C, ^l^ LDL-C, HbA1C	Mobile apps, personalized messages, coaching, step tracker	[31]
Eppes, E.V., 2023	SR	RCTs, pre-post studies, pilot studies, feasibility study, prospective cohort study, descriptive study, cross-sectional study	USA	Parents of young children and adolescents (age range not specified)	2009–2022	5	Health promotion	^m^ FV consumption, healthy diet, healthy weight	Mobile apps, messaging tools, app and wearable device	[32]
De Santis, K.K., 2023	SR	Primary study, SR, RCT, Non-randomized studies	Germany	50–99 years	2005–2022 (June)	4	Health promotion and disease prevention	Health promotion, mobility, mental health, nutrition, or cognition	Website, text-message, email, app, exergaming, mobile phone	[33]
Chew, H.S.J., 2023	SR, MA	RCTs	Singapore	Adults (>18 years)	1900–2022	7	Health promotion and weight loss intervention	Weight loss	Mobile apps and coaching	[34]
Giurgiu, M., 2023	SR, MA	Validation studies	Germany	≥18 years	1970–2020 (December)	5	Evaluation of the characteristics, validity, and quality of wearable devices used for 24 h for the measurement of anamnestic parameters	Sleep patterns, postures, sedentary behavior, physical activity, energy expenditure, steps count	Wearable device: wearing position, software, epoch-length, algorithm/cut-point	[35]
Shoneye, C.L., 2023	SR	RCTs	Australia	≥18 years	1990–2020	5	Diet quality	Dietary feedback, tailored weight-loss interventions, digital weight loss intervention	Mobile app, website, accelerometer, computer software, text message	[36]

Abbreviations: ^a^ SR, systematic review; ^b^ MA, meta-analysis; ^c^ RCT, Randomized Controlled Trial; ^d^ BMI, Body Mass Index; ^e^ LIPA, Light-Intensity Physical Activity; ^f^ PA: Physical Activity; ^g^ CVD, cardiovascular disease; ^h^ HbA1c, Hemoglobin A1c; ^i^ FPG, Fasting Plasma Glucose; ^j^ VO_2_max, maximal oxygen consumption; ^k^ HDL-C, High-Density Lipoprotein Cholesterol; ^l^ LDL-C, Low-Density Lipoprotein Cholesterol; ^m^ FV, fruit and vegetable.

**Table 2:** AMSTAR 2 checklist and corresponding score of the studies included in the Scoping Review.

Authors, Year (Reference)	1. PICO	2. Review Methods *	3. Study Selection	4. Search Strategy *	5. Study Selection	6. Data Extraction	7. Excluded Studies *	8. Describe Studies	9. ROB Tool *	10. Report Funding	11. Statistical Methods *	12. ROB Assessment	13. ROB Discussion *	14. Study Differences	15. Pubblication Bias *	16. COI and Funding	AMSTAR 2 Quality Rating
Mateo, G.F. et al., 2015 [14]	Yes	Yes	Yes	Partial Yes	Yes	Yes	Yes	Partial Yes	Partial Yes	No	Yes	Yes	Yes	Yes	Yes	Yes	Moderate
Semper H.M. et al., 2016 [15]	Yes	Partial Yes	Yes	Partial Yes	Yes	Yes	Yes	Partial Yes	Partial Yes	No	N/A	N/A	Yes	Yes	N/A	No	Low
Schoeppe S. et al., 2016 [16]	Yes	Partial Yes	Yes	Partial Yes	Yes	Yes	Yes	Partial Yes	No	No	N/A	N/A	No	Yes	N/A	Yes	Critically Low
Füzéki E et al., 2017 [17]	Yes	Partial Yes	No	Partial Yes	Yes	Yes	No	Partial Yes	No	No	N/A	N/A	No	Yes	N/A	Yes	Critically Low
Heesch K.C. et al., 2018 [18]	No	Partial Yes	No	Partial Yes	Yes	Yes	No	Partial Yes	No	Yes	N/A	N/A	Yes	Yes	N/A	Yes	Low
Lee M. et al., 2018 [19]	Yes	Partial Yes	Yes	Partial Yes	Yes	Yes	Yes	Partial Yes	Partial Yes	No	N/A	N/A	Yes	Yes	N/A	Yes	Low
Maddison R. et al., 2019 [8]	Yes	Partial Yes	Yes	Yes	Yes	Yes	Yes	Partial Yes	No	Yes	N/A	N/A	No	No	N/A	Yes	Low
Mandracchia F. et al., 2019 [7]	Yes	Partial Yes	Yes	Partial Yes	Yes	Yes	Yes	Partial Yes	Partial Yes	No	N/A	N/A	Yes	Yes	N/A	Yes	Low
Villinger K. et al., 2019 [6]	Yes	Partial Yes	Yes	Yes	Yes	Yes	Yes	Partial Yes	Partial Yes	No	Yes	Yes	Yes	Yes	Yes	Yes	Moderate
Zarnowiecki D. et al., 2020 [20]	Yes	Partial Yes	Yes	Partial Yes	Yes	Yes	Yes	Partial Yes	No	No	Partial Yes	No	Yes	No	No	Yes	Critically Low
Paramastri R. et al., 2020 [21]	Yes	Partial Yes	Yes	Partial Yes	Yes	Yes	No	Partial Yes	No	No	N/A	N/A	Yes	No	N/A	Yes	Critically Low
Hall K.S. et al., 2020 [22]	Yes	Partial Yes	Yes	Partial Yes	Yes	Yes	No	Partial Yes	No	Yes	N/A	N/A	No	Yes	N/A	Yes	Critically Low
Cavero-Redondo I. et al., 2020 [23]	Yes	Yes	Yes	No	Yes	Yes	Yes	Partial Yes	No	No	Yes	Yes	Yes	Yes	Yes	Yes	Low
McDonough D.J. et al., 2021 [24]	Yes	Partial Yes	Yes	Partial Yes	Yes	Yes	Partial Yes	Partial Yes	Yes	No	Yes	Yes	Yes	Yes	Yes	Yes	Moderate
Dixit S. et al., 2021 [4]	Yes	Partial Yes	Yes	Partial Yes	Yes	Yes	No	Partial Yes	No	No	No	No	No	No	No	Yes	Critically Low
Robert C. et al., 2021 [25]	Yes	Partial Yes	Yes	Partial Yes	Yes	Yes	Yes	Partial Yes	Partial Yes	No	Yes	Yes	No	No	Yes	Yes	Low
Davies A. et al., 2021 [26]	Yes	Yes	Yes	Partial Yes	Yes	Yes	Yes	Partial Yes	No	Yes	N/A	N/A	No	Yes	N/A	Yes	Low
Raber M. et al., 2021 [27]	Yes	Partial Yes	Yes	Partial Yes	Yes	Yes	Yes	Partial Yes	Partial Yes	No	N/A	N/A	Yes	Yes	N/A	Yes	Low
Chevance G. et al., 2022 [28]	Yes	Partial Yes	No	No	Yes	Yes	Partial Yes	Partial Yes	Yes	Yes	Yes	Yes	Yes	Yes	Yes	Yes	Moderate
Scarry A. et al., 2022 [29]	Yes	Partial Yes	Yes	Partial Yes	Yes	Yes	Yes	Partial Yes	Partial Yes	No	N/A	N/A	Yes	Yes	N/A	Yes	Low
Ferguson T. et al., 2022 [30]	Yes	Partial Yes	Yes	Partial Yes	Yes	Yes	No	Yes	Yes	No	Yes	Yes	Yes	Yes	Yes	Yes	Moderate
Chew H.S.J. et al., 2022 [31]	Yes	Partial Yes	Yes	Partial Yes	Yes	Yes	Yes	Yes	Partial Yes	Yes	Yes	Yes	Yes	Yes	Yes	Yes	Moderate
Eppes E.V. et al., 2023 [32]	Yes	Partial Yes	Yes	Partial Yes	Yes	Yes	Yes	Partial Yes	Partial Yes	No	N/A	N/A	Yes	Yes	N/A	Yes	Low
De Santis K.K. et al., 2023 [33]	No	No	Yes	Partial Yes	Yes	Yes	No	Partial Yes	No	Yes	N/A	N/A	No	Yes	N/A	Yes	Critically Low
Chew H.S.J. et al., 2023 [34]	Yes	Yes	Yes	Partial Yes	Yes	Yes	Yes	Partial Yes	Partial Yes	No	Yes	Yes	Yes	No	Yes	Yes	Moderate
Giurgiu M. et al., 2023 [35]	Yes	Partial Yes	No	Yes	Yes	Yes	No	Partial Yes	Yes	No	N/A	N/A	Yes	Yes	N/A	Yes	Moderate
Shoneye C.L. et al., 2023 [36]	Yes	Partial Yes	Yes	Partial Yes	No	No	Yes	Yes	Partial Yes	No	N/A	N/A	Yes	Yes	N/A	Yes	Critically Low

Abbreviations: N/A, not applicable. AMSTAR 2 checklist: 1. Did the research questions and inclusion criteria for the review include the components of PICO? 2. * Did the report of the review contain an explicit statement that the review methods were established prior to the conduct of the review and did the report justify any significant deviations from the protocol? 3. Did the review authors explain their selection of the study designs for inclusion in the review? 4. * Did the review authors use a comprehensive literature search strategy? 5. Did the review authors perform study selection in duplicate? 6. Did the review authors perform data extraction in duplicate? 7. * Did the review authors provide a list of excluded studies and justify the exclusions? 8. Did the review authors describe the included studies in adequate detail? 9. * Did the review authors use a satisfactory technique for assessing the risk of bias (RoB) in individual studies that were included in the review? Different answers for RCT or NRSI. 10. Did the review authors report on the sources of funding for the studies included in the review? 11. * If meta-analysis was performed did the review authors use appropriate methods for statistical combination of results? Different answers for RCT or NRSI. 12. If meta-analysis was performed, did the review authors assess the potential impact of RoB in individual studies on the results of the meta-analysis or other evidence synthesis? 13. * Did the review authors account for RoB in individual studies when interpreting/discussing the results of the review? 14. Did the review authors provide a satisfactory explanation for, and discussion of, any heterogeneity observed in the results of the review? 15. * If they performed quantitative synthesis did the review authors carry out an adequate investigation of publication bias (small study bias) and discuss its likely impact on the results of the review? 16. Did the review authors report any potential sources of conflict of interest, including any funding they received for conducting the review? *: AMSTAR 2 critical domains are marked with asterisk.

**Table 3:** AMSTAR 2 checklist responses by domain focusing on critical domains marked with asterisk.

Responses by Domain	1.	2. *	3.	4. *	5.	6.	7. *	8.	9. *	10.	11. *	12.	13. *	14.	15. *	16.
Yes	25	4	23	3	26	26	17	3	4	7	9	9	19	21	9	26
Partial Yes	0	22	0	22	0	0	2	24	12	0	1	0	0	0	0	0
No	2	1	4	2	1	1	8	0	11	20	1	2	8	6	2	1
N/A	0	0	0	0	0	0	0	0	0	0	16	16	0	0	16	0

Abbreviations: N/A, not applicable. (*) Domains considered critical according to the AMSTAR 2.

## Supplementary Materials

The following supporting information can be downloaded at: https://www.mdpi.com/article/10.3390/nu17223542/s1. Table S1: PRISMA 2020 Checklist of Reporting Items; File S1: Search strategy for Umbrella Review.

## Abbreviations

SR	Systematic Review
MA	Meta-Analysis
RCT	Randomized Controlled Trial
AMSTAR	A MeaSurement Tool to Assess systematic Reviews
BCT	Behavioral change techniques
BMI	Body Mass Index
LIPA	Light-Intensity Physical Activity
PA	Physical Activity
CVD	Cardiovascular Disease
HbA1	Hemoglobin A1c
FPG	Fasting Plasma Glucose
VO_2_max	Maximal Oxygen Consumption
HDL-C	High-Density Lipoprotein Cholesterol
LDL-C	Low-Density Lipoprotein Cholesterol
FV	Fruit and Vegetable
TBP	Theory-Based Podcast
