# Supplementary material for: Empowering Health Through Digital Lifelong Prevention: An Umbrella Review of Apps and Wearables for Nutritional Management
Source: Nutrients. 2025 Nov 12;17(22):3542. doi: 10.3390/nu17223542 (PMC12655027; doi:10.3390/nu17223542)
Supplement: Supplementary file 1 [file nutrients-17-03542-s001.zip › File S1.pdf]

Search strategy for Umbrella Review

Searches

The PubMed/Medline, Scopus and EBSCO databases will be searched.

Filters: English language, Meta-Analysis, Reviews, SRs, 10 years, date range.

First step

<u>PubMed/Medline</u>

(((food intake[Title/Abstract] OR food behaviour[Title/Abstract] OR nutrition behaviour[Title/Abstract] OR diet[Title/Abstract] OR nutrition[Title/Abstract] OR nutritional habits[Title/Abstract] OR nutritional database[Title/Abstract] OR healthy nutrition[Title/Abstract] OR healthy food[Title/Abstract]) AND (digital device[Title/Abstract] OR digital technology[Title/Abstract] OR digital tool[Title/Abstract] OR portable device[Title/Abstract] OR wearable device[Title/Abstract] OR mobile device[Title/Abstract] OR sensor[Title/Abstract] OR accelerometer[Title/Abstract] OR mobile app[Title/Abstract] OR smartphone[Title/Abstract] OR smartwatch[Title/Abstract] OR watch[Title/Abstract] OR phone[Title/Abstract] OR bracelet[Title/Abstract] OR earphone[Title/Abstract] OR app[Title/Abstract] OR application[Title/Abstract] OR digital app[Title/Abstract] OR mobile health app[Title/Abstract] OR software[Title/Abstract] OR mHealth[Title/Abstract] OR eHealth[Title/Abstract] OR Prototyping wearable device[Title/Abstract] OR digital nutrition[Title/Abstract] OR smart device[Title/Abstract] OR digital tool[Title/Abstract] OR mobile application[Title/Abstract] OR wearable technologies[Title/Abstract] OR pedometers[Title/Abstract]) AND (valid*[Title/Abstract] OR reproducib*[Title/Abstract] OR reliab*[Title/Abstract] OR monitor*[Title/Abstract] OR tracking[Title/Abstract] OR surveillance[Title/Abstract] OR efficac*[Title/Abstract] OR assess*[Title/Abstract] OR evaluat*[Title/Abstract]))

<u>Scopus</u>

(food AND intake[title/abstract] OR diet[title/abstract] OR nutrition[title/abstract] OR nutritional AND habits[title/abstract] OR healthy AND nutrition[title/abstract] OR healthy AND food[title/abstract]) AND (wearable AND device[title/abstract] OR mobile AND device[title/abstract] OR sensor[title/abstract] OR accelerometer[title/abstract] OR mobile AND app[title/abstract] OR smartphone[title/abstract] OR smartwatch[title/abstract] OR watch[title/abstract] OR mobile AND health AND app[title/abstract] OR wearable AND technologies[title/abstract] OR pedometers[title/abstract]) AND (valid*[title/abstract] OR reproducib*[title/abstract] OR reliab*[title/abstract] OR monitor*[title/abstract] OR tracking[title/abstract] OR surveillance[title/abstract] OR efficac*[Title/Abstract] OR assess*[title/abstract] OR evaluat*[title/abstract]) AND PUBYEAR > 2013 AND PUBYEAR < 2024

AND (LIMIT-TO (EXACTKEYWORD , "Review") OR LIMIT-TO (EXACTKEYWORD , "Systematic Review"))

EBSCO

AB (diet OR nutrition OR nutritional habits OR healthy nutrition OR healthy food) AND AB (mobile app OR smartphone OR smartwatch OR wearable device OR digital nutrition OR smart device OR digital tool OR wearable technologies OR pedometers) AND AB (valid OR reproducible OR efficacy OR reliable OR monitor OR tracking OR surveillance OR assess OR evaluate) AND AB (systematic review OR meta-analysis)

Second step

PubMed

(food intake[Title/Abstract] OR food behavior[Title/Abstract] OR diet*[Title/Abstract] OR nutrition*[Title/Abstract]) AND (app*[Title/Abstract] OR mobile app*[Title/Abstract] OR smartphone app*[Title/Abstract] OR smartwatch[Title/Abstract])) OR tablet app*[Title/Abstract])) AND (tracking[Title/Abstract] OR surveillance[Title/Abstract] OR assess*[Title/Abstract] OR evaluat*[Title/Abstract] OR effectiv*[Title/Abstract] OR efficacy[Title/Abstract])

(food intake[Title/Abstract] OR food behavior[Title/Abstract] OR diet*[Title/Abstract] OR nutrition*[Title/Abstract]) AND (digital device[Title/Abstract] OR digital technology[Title/Abstract] OR portable device[Title/Abstract] OR wearable device[Title/Abstract] OR mobile device[Title/Abstract] OR accelerometer[Title/Abstract] OR smartwatch[Title/Abstract] OR watch[Title/Abstract] OR bracelet[Title/Abstract] OR earphone[Title/Abstract] OR sensor[Title/Abstract] OR prototyping wearable device[Title/Abstract] OR smart device[Title/Abstract] OR digital tool[Title/Abstract] OR wearable technologies[Title/Abstract] OR pedometers[Title/Abstract]) AND (tracking[Title/Abstract] OR surveillance[Title/Abstract] OR assess*[Title/Abstract] OR evaluat*[Title/Abstract] OR effectiv*OR efficacy[Title/Abstract])

Scopus

(TITLE-ABS-KEY ((food AND intake OR food AND behavior OR diet OR nutrition)) AND TITLE-ABS-KEY ((app OR mobile AND app OR smartphone AND app OR smartwatch OR tablet AND app)) AND TITLE-ABS-KEY ((tracking OR surveillance OR assess OR evaluate OR effective* OR efficacy))) AND PUBYEAR > 2013 AND PUBYEAR < 2024 AND (LIMIT-TO (EXACTKEYWORD , "Review") OR LIMIT-TO (EXACTKEYWORD , "Systematic Review")) AND (LIMIT-TO (DOCTYPE , "re")) AND (LIMIT-TO (LANGUAGE , "English"))

(TITLE-ABS-KEY ((diet OR nutrition OR nutritional AND habits OR healthy AND nutrition OR healthy AND food)) AND TITLE-ABS-KEY ((wearable AND device OR smart AND device OR

wearable AND technologies OR accelerometer)) AND TITLE-ABS-KEY ((valid OR reproducible OR efficacy OR reliable OR monitor OR tracking OR surveillance OR assess OR evaluate)) AND TITLE-ABS-KEY ((systematic AND review OR meta-analysis)) AND PUBYEAR > 2013 AND PUBYEAR < 2024 AND (LIMIT-TO (EXACTKEYWORD , "Review") OR LIMIT-TO (EXACTKEYWORD , "Systematic Review"))

EBSCO

AB (food intake OR food behavior OR diet OR nutrition) AND AB (app OR mobile app OR smartphone app OR smartwatch OR tablet app) AND AB (tracking OR surveillance OR assess OR evaluate OR effective* OR efficacy) AND AB (systematic review OR meta-analysis review)

AB (diet OR nutrition OR nutritional habits OR healthy nutrition OR healthy food) AND AB (wearable device OR smart device OR digital tool OR wearable technologies OR pedometers) AND AB (valid OR reproducible OR efficacy OR reliable OR monitor OR tracking OR surveillance OR assess OR evaluate) AND AB (systematic review OR meta-analysis)
